# Pulmonary Abscess, Containing a Large Amount of Matter, Cured by an Operation

**Published:** 1856-06

**Authors:** Alfred M. Leonard

**Affiliations:** Clarkson, Monroe County, N. Y.


ART. II.—*Pulmonary Abscess, containing a large amount of Matter, cured by an Operation.* By ALFRED M. LEONARD, M. D., Clarkson, Monroe County, N. Y.

Although the case I am about to present is of some time standing, perhaps it will be no less interesting.

In the early part of June, 1852, I was called to visit a young man by the name of Joseph Lafountain, who had lately returned from a southern county where he had been on a visit, and had been confined there sick some three months, laboring under symptoms of Phthisis. When I was called to see him after his return to this place, I found him unable to walk the room; he was attended with a colliquative diarrhœa; pain in the left side; irregular febrile action; flushed cheeks, and a profuse perspiration on going to sleep; pulse about 100, quick and hard, though easily compressed; tongue somewhat coated, of a brownish yellow cast or appearance; severe cough, and expectoration of a muco-purulent matter.

I apprised himself and his friends of the nature of the case, as I viewed it to be a case of phthisis, and having passed the meridian, offered but little or no prospect of improvement or probability of cure. He was not alarmed in the least at this explanation, and said he only expected palliative treatment; he had no expectation of a cure.

However, as there was nothing apparently to lose in the case, I presumed to prescribe and wait for the effects to dictate for the future. My first prescription consisted of a blister to the back between the shoulders, covering the most of the dorsal vertebræ. I then gave him small portions of calomel, about 6 grs. each, combined with 1 gr. tannin, $\frac{1}{4}$ gr. morphine, and about the twelfth of a grain of tart. antimony, to be given night and morning, with a view to correct the biliary secretions, as well also as the secretions in general.

My further prescription at the time, was as follows:

> ℞ Tr. sanguin, tr. ipp., āā one ounce,
> Quinine, ℈i.,
> Morphine, grs. x,
> Capsicum and sal. tart. also 10 grs. each, all to be combined and given in such quantity as the stomach would bear, four times a day, and to be taken in a little weak brandy and water.

I also left a few powders of tannin, about one grain each, to be taken, if necessary, to control the diarrhœa.

I continued him under this treatment about four days, when signs of improvement began to manifest themselves. The diarrhœa became controlled, and the discharges were less frequent and of a biliary cast. Tongue began to clear off; fever much abated; perspiration but little; cough and expectoration improved; pulse soft and less frequent. Under this state of things I had confidence to continue my prescription, with some few alterations. I omitted the calomel (in its prescription) and substituted the bichloride of mercury, which I united with the preparation of sanguinaria, &c., and omitted in that the sal. tart. This was continued for about a week, and the patient continuing to improve I thought proper to omit a further use of the mercury. It was, therefore, omitted, and the sal. tart. again substituted in its place as at first, and the prescription continued.

At this time the patient began to walk about the room, and to sit up the most of the time at short intervals. At this time, thinking that he was laboring under debility, more than any active disease, I directed for him an infusion of gentian, colombo, and camomile flos., to be drank freely through the day; guarded the bowels with gentle cathartics, and about every three days a blue pill, and at the end of four weeks I dismissed him, his primary symptoms having all left him, and he was able to walk the streets at pleasure, and continued in tolerable health till the fore part of June following, with only slight and occasional pain in the left side, which was so slight that he had not thought it necessary to ask any advice. At this time the pain became more continuous and severe in the left side, and seemed to involve the whole of the left lung in a deep-seated and heavy pain.

This state of things continued to increase, although his strength did not seem to suffer as much as might have been expected, still it was evident that a disease of some extent and importance was preying on the vitality of the system; his strength began to fail, although the cough and expectoration were very trifling and fever very slight.

In the early part of July he became unable to walk about but little, and complained of a swelling situated on the left side of the vertebræ and directly under the last or lower rib. About the middle of July I was called upon to visit him, and then learned from the family the above history of his case, as I had not prescribed to him since I dismissed him the season before.

On my visit to him, which was in the evening, I found him lying on a lounge upon his right side; and upon inquiry he told me he could not lie on the left side, because of a swelling on that side, and he thought it would have to be opened. His fever was very trifling, and there seemed to be but little disturbance in the system further than a general debility.

I therefore proceeded to examine the swelling, and at first did not find much of a swelling, although a slight elevation, whose base was about three inches in diameter, and upon pressure gave the appearance of fluctuation, and upon laying my hand on it and pressing its surface generally, it would recede and again return by removing the pressure. I then pressed it back and asked him to inspire, which he did, and it immediately filled to its full size. I then apprised the patient that the swelling was nothing more or less than a portion of an abscess, more or less connected with the lung of that side, and probably within the pleura pulmonalis. I then explained to the patient and friends the importance and effect of the operation, if the disease was as I expected, that if the abscess were connected with the lung to any considerable extent, and the right lung involved in disease so far as to fail to do its office of inspiration and expiration, it was a great doubt if he could survive the operation. I therefore postponed the operation till morning, preferring day-light to meet the consequences as they might prove.

Accordingly, I called on him the next morning and circumstances confirmed my previous opinion. In the presence of several gentlemen I then proceeded to operate, having previously provided the necessary stimulants that the case might require.

I commenced the operation by making an incision opposite and about three inches to the left of the body of the last dorsal vertebra, and about under the posterior angle of the rib. I chose this situation as the angle allowed the abscess to approximate nearer the surface and opposite the body of the vertebra, that I might not endanger the nerve and artery thrown out between the vertebræ. My first incision I made about an inch or more deep, with my abscess lance, and about an inch in length, endeavoring to pass through the cellular tissue and external muscles.

With my finger introduced into the incision I was able to feel more distinctly the situation of the abscess. I then took my lance-bladed trochar, and directing it upward and inward, or a little to the right I pressed it in that direction till I was satisfied it had reached the cavity of the thorax, I then allowed the canula to remain, and withdrew the trochar, which was followed by a current of pus, which being received in a vessel for the purpose, was allowed to discharge till it had discharged the amount of five quarts, when the patient began to receive and discharge air through the orifice, and immediately swooned away. I immediately closed the orifice with my fingers, and by the use of stimulants both diffusible and permanent, he soon recovered sufficiently to carry on a tolerable respiration.

The orifice was then closed with a tent and adhesive straps, and the patient allowed to go to bed, and rested comfortably through the day and following night.

The next day I found him sitting up and comfortable. I opened the orifice and drew away one quart of pus, but with very little effect on the respiration.

I then, for further satisfaction, introduced a long probe for the purpose of ascertaining in a measure the extent of the abscess. I directed my probe in the same direction as I did the trochar, and passed it, without any obstruction, to the vicinity of the sterno-clavicular articulation, and the pulsations of the heart were distinctly felt by the probe. I then dressed the wound as before, and put the patient upon nourishing diet, with brandy and water, and about one grain of quinine once in about six hours; kept the bowels loose by gentle laxatives; and he continued to improve, the discharge grew less, and in two weeks was in nowise troublesome, and needed but a compress and bandage, and at the end of four weeks he was so far recovered as to be able to walk about the house and yards. After this I only saw him occasionally, giving him some tonics, and he soon became able to walk about the village and do some labor, and recovered tolerable health. His health continued to be very comfortable during two years, when he was taken sick with a severe attack of bilious pneumonia, and I being at Albany at the time of his sickness, did not see him, and he died after a short illness, and as I was absent at the time of his death, I had not the opportunity of a post-mortem examination.

